# Neuromodulation by surprise: a biologically plausible model of the learning rate dynamics

**DOI:** 10.1186/1471-2202-15-S1-P159

**Published:** 2014-07-21

**Authors:** Mohammad Javad Faraji, Kerstin Preuschoff, Wulfram Gerstner

**Affiliations:** 1 School of Life Sciences, Brain Mind Institute and School of Computer and Communication Sciences, Ecole Polytechnique Federal de Lausanne, Lausanne, Swizerland

## 

Surprise is a central concept in learning, attention and the study of the neural basis of behaviour. However, how surprise affects learning and more specifically, how surprise affects synaptic learning rules in neural networks is largely undetermined. Here we study how surprise facilitates learning in different environments and how surprise can potentially modulate Hebbian learning in the form of a global factor in multi-factor learning rules.

**Figure 1:**
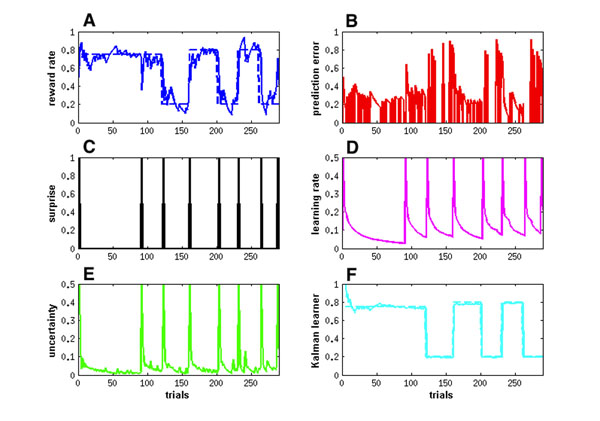
Estimation of the probability of reward delivery in a reversal task. **A**. Estimated reward rate. **B**. Reward prediction error. **C**. Surprise measure. **D**. Learning rate. **E**. Uncertainty measure. **F**. Optimal Kalman learner.

Learning rate is a crucial factor in determining to what extent the learning agent should rely on the newly acquired information rather than the old information in building its own internal model of the external world. Both theory and empirical evidences suggest that the learning rate should be adjusted under different circumstances for having an optimal and effective learning strategy. We propose a simple and biologically plausible model that describes the dynamics of the learning rate in terms of surprise and uncertainty measures. We apply our model to three different tasks: a reversal task (Fig. 1), a dynamic decision making task, and a dynamic clustering task.

Our proposed model explains how the agent should effectively control the speed of learning in different environments such that it matches both theory and empirical evidences from human and animal subjects. This model explains why surprising events provoke humans and animals to learn faster and why they rapidly adapt to changing environments. It also addresses the question of what the effective learning rate should be in both stable (either low-risky or high-risky) and volatile environments. Here effectiveness is defined as having a higher accuracy in learning a task, for instance the estimation of the mean reward in classic reinforcement learning, for a given time and computational complexity as well as the available memory as our constraints. This study also suggests a functional connectivity pattern for the neurochemical systems that are related to contextual modulation of learning rate. Further, it explains why we need different neuromodulators with distinct functional roles to act in parallel in a broad range of distribution and proposes suitable candidates responsible for measuring different quantities we need in the model.

